# P006 Treatment options if burosumab significantly improves symptoms related to X-linked hypophosphatemia but causes massive injection site reactions in adults

**DOI:** 10.1093/rap/rkag113.036

**Published:** 2026-11-03

**Authors:** Fiona Kirkham-Wilson, Siraj Misbah, Kassim Javaid

**Affiliations:** University of Oxford, Oxford, United Kingdom; Oxford University Hospitals, Oxford, United Kingdom; University of Oxford, Oxford, United Kingdom

## Abstract

**Introduction:**

X-linked hypophosphatemia (XLH) is a rare metabolic bone disease caused by a mutation in the PHEX gene leading to elevated levels of FGF23 and associated low phosphate. People with XLH have a propensity to pseudofractures, dental abscesses, and other complications. Burosumab is a monoclonal antibody which binds FGF23 and can normalise the phosphate level. In the UK, every adult with XLH can receive burosumab, and many patients have found their lives revolutionised. Here, a case of an immediate onset skin reaction with urticarial response following injection is discussed, along with the treatment plan to allow the patient to continue burosumab.

**Case description:**

Patient AB is a 46-year-old woman, diagnosed with XLH aged nine. Her mother became a wheelchair user secondary to XLH. AB’s son also has XLH. When burosumab became available to the paediatric population, AB’s son started burosumab: he had no side effects and has achieved a near-normal height. AB attended XLH family day (an event for people in the UK affected by XLH) in mid-2025, and decided to start burosumab. Treatment started in late 2025.

Prior to burosumab, AB was taking 800iu of vitamin D a day (having stopped phosphate replacement and activated vitamin D in 2022); she was experiencing stiffness in her lower limbs and shoulders, pain in her left knee and back, and she had extensive dental involvement. Her PTH was 13.7 pmol/L (normal range 3-12pmol/L) and phosphate was 0.43mmol/L (normal range 0.7-1.45mmol/L). She had a pseudofracture of her right femur in 2019.

After starting 70mg burosumab every 4 weeks, AB noted improvement: her pain decreased (stiffness remained unchanged) and she has not had dental abscesses. AB has started exercise classes and began losing weight. Her phosphate normalised to 0.82mmol/L. After her third dose of burosumab, AB developed a hot, red, tender area of inflammation around the injection site. Cetirizine 10mg and hydrocortisone cream was started to facilitate the fifth dose, but the reactions continued: after dose seven the inflammatory wheal was more than 15cm in diameter, with prevalent pruritus, causing AB to seek medical help. Ceterizine was increased to 20mg twice daily for 48 hours before the injection. Injection site reactions are a known side effect of burosumab, however, such a significant reaction prompted referral to immunology. Using skin prick testing, it was possible to confirm AB was experiencing a reaction secondary to sensitisation to burosumab, likely IgE mediated. She currently continues burosumab with cetirizine.

**Discussion:**

Ideally the burosumab would have been stopped or switched to activated vitamin D and phosphate, but given her positive response to burosumab, it has been agreed by the immunology, metabolic bone teams and the patient to continue with antihistamine and steroid cover with safety netting if the patient develops systemic allergic symptoms, including an inpatient desensitisation treatment.

To date the reaction to the burousmab has not affected the biochemical or general clinical response and this algorithm may be of value for other patients with XLH on burosumab developing severe injection site reactions.

**Key learning points:**

1. The scale of benefit fromburosumabby adults livingwith XLH led to an escalation of measures to minimise the injection site reactionsin conjunction with immunology.

2. Patch testing wasrequiredto confirm the nature of the injection site reaction.

3. A combination of high dose anti histamine and oral prednisolone of X days was effective insignificantlyreducing the allergic reaction.

4. Safety netting isrequiredin case of asystemic allergic reaction.

5. Shareddecision-makingapproach betweenpatients and multi-disciplinaryteamhighlightshow rare bone disorders can be safely managed in cases where very few treatments are available.

